# Persistent hypercoagulability in dogs envenomated by the European adder (*Vipera berus berus*)

**DOI:** 10.1371/journal.pone.0263238

**Published:** 2022-02-18

**Authors:** Hannah J. Harjen, Marit Hellum, Runa Rørtveit, Malin Oscarson, Kristin P. Anfinsen, Elena R. Moldal, Susanna Solbak, Sandip M. Kanse, Carola E. Henriksson

**Affiliations:** 1 Faculty of Veterinary Medicine, Department of Companion Animal Clinical Sciences, Norwegian University of Life Sciences, Oslo, Norway; 2 Institute of Clinical Medicine, University of Oslo, Oslo, Norway; 3 The Blood Cell Research Group, Department of Medical Biochemistry, Oslo University Hospital, Oslo, Norway; 4 Faculty of Veterinary Medicine, Department of Preclinical Sciences and Pathology, Norwegian University of Life Sciences, Oslo, Norway; 5 Norwegian Food Safety Authority, Brumunddal, Norway; 6 Anicura Dyresykehus Oslo, Oslo, Norway; 7 Anicura Jeløy Dyresykehus, Moss, Norway; 8 Institute of Basic Medical Sciences, University of Oslo, Oslo, Norway; Institute of Experimental Hematology and Transfusion Medicine, University Clinic of Bonn, GERMANY

## Abstract

**Background:**

Envenomation by the European adder, *Vipera berus berus (Vbb)*, is a medical emergency. The overall *in vivo* haemostatic effects of pro- and anticoagulant components in *Vbb* venom, and the downstream effects of cellular injury and systemic inflammation, are unclear.

**Objectives:**

To longitudinally describe the global coagulation status of dogs after *Vbb* envenomation and compare to healthy controls. A secondary aim was to investigate differences between dogs treated with and without antivenom.

**Methods:**

Citrated plasma was collected at presentation, 12 hours (h), 24 h, 36 h and 15 days after bite from 28 dogs envenomated by *Vbb*, and from 28 healthy controls at a single timepoint. Thrombin generation (initiated with and without exogenous phospholipids and tissue factor), thrombin-antithrombin (TAT)-complexes and the procoagulant activity of phosphatidylserine (PS)-expressing extracellular vesicles (EVs), expressed as PS-equivalents, were measured.

**Results:**

At presentation the envenomated dogs were hypercoagulable compared to controls, measured as increased thrombin generation, TAT-complexes and PS-equivalents. The hypercoagulability decreased gradually but compared to controls thrombin generation and PS-equivalents were still increased at day 15. The discrepancy in peak thrombin between envenomated dogs and controls was greater when the measurement was phospholipid-dependent, indicating that PS-positive EVs contribute to hypercoagulability. Lag time was shorter in non-antivenom treated dogs, compared to antivenom treated dogs <24 h after envenomation.

**Conclusions:**

Hypercoagulability was measured in dogs up to 15 days after *Vbb* envenomation. Dogs treated with antivenom may be less hypercoagulable than their non-antivenom treated counterparts. Thrombin generation is a promising diagnostic and monitoring tool for *Vbb* envenomation.

## Introduction

The European adder, *Vipera berus berus (Vbb)*, is a medically important snake, responsible for approximately 60% of all *Vipera* species envenomations in humans in Europe [1, 2]. Dogs are more commonly bitten by *Vbb* than humans, and as such provide a natural, accessible model for the effects of envenomation. Since *Vbb* is the only venomous snake in Scandinavia [2, 3], clinical signs of snake envenomation in these countries can be attributed to this species alone. Pain, oedema, nausea, arrhythmia, acute kidney injury and coagulopathy have been reported in humans and dogs [1, 4–9], but studies of *Vbb* envenomation are limited, and the clinical consequences of envenomation by this species are not fully known. Case reports have described limb and pulmonary thrombosis in humans [6, 10] and microthrombi in the renal vasculature of dogs [11] bitten by *Vbb*. However, persistent bleeding from the snakebite wound, haematomas and ecchymoses have also been reported in dogs and humans bitten by this species of snake [4, 12, 13].

*Vbb* venom contains both pro- and anti-coagulant components such as phospholipase A_2_ (PLA_2_)_,_ thrombin-like enzymes, factor X (FX)-activating enzymes and platelet aggregate inhibitors [14–17], the net effect of which is not known. In addition to direct effects of venom components, downstream effects of systemic inflammation and cellular injury, including shedding of extracellular vesicles (EVs) may also lead to clinical and laboratory coagulation abnormalities. EVs are cell-derived membrane structures originating either from the endosomal system or from the plasma membrane of activated or apoptotic cells [18]. When EVs are shed from the plasma membrane, phosphatidylserine (PS), a negatively charged phospholipid normally localised to the inner leaflet of the membrane, is externalised. This negatively charged phospholipid surface of the EVs provide a suitable surface for the assembly of blood coagulation factors. In addition, tissue factor (TF), the main initiator of coagulation may be present on EVs, making them circulating entities able to initiate and propagate thrombin generation systemically.

Coagulation analyses may help identify individuals at risk of haemorrhage or thromboembolic disease and thereby contribute to appropriate treatment, but studies reporting such analyses after *Vbb* envenomation are sparse and contradictory. Studies in humans and dogs have shown thrombocytopenia, prolonged prothrombin time (PT)/international normalized ratio (INR), and prolonged activated partial thromboplastin time (APTT), but hypercoagulability indicated by decreased INR and increased D-dimer concentrations has also been reported during the first 36 hours after *Vbb* bite [4, 12, 19]. Longitudinal studies assessing the time course of coagulation disturbances after envenomation by *Vbb* as well as the effect of antivenom treatment, are lacking.

Thrombin generation assays such as the calibrated automated thrombogram (CAT) have proven useful to assess the *in vitro* thrombin-generating capacity of venoms, and the efficacy of antivenoms [20, 21]. One study evaluating in vitro haemostatic changes associated with Brazilian snake venoms, concluded that PT and APTT were only able to detect mild hypocoagulable states, whilst thrombin generation measured by CAT detected both hypo- and hypercoagulability [20]. Measurement of thrombin generation may therefore give a more complete picture of the global coagulation status after envenomation than routine clotting analyses. However, to our knowledge, measurements of thrombin generation have not been performed in humans or dogs after envenomation by any snake species.

The calibrated automated thrombogram assesses net thrombin generation under the influence of procoagulant and anticoagulant forces. The use of various trigger reagents containing different concentrations of phospholipid (PL) and TF in the CAT assay allows evaluation of the different pro- and anticoagulant contributors to the thrombin generation potential, such as venom and EVs [22]. CAT can be complemented by the measurement of PS activity to further evaluate EV-associated procoagulant activity, and thrombin-antithrombin complexes (TAT) as an indicator of in vivo thrombin generation [23].

The primary aim of this study was to serially assess the global coagulation status of dogs envenomated by *Vbb*, compared to a group of healthy control dogs. We measured thrombin generation (initiated with and without exogenous PL, and TF), TAT complexes and the phospholipid-dependent activity of PS-exposing EVs. A secondary aim was to investigate differences in the global coagulation status of dogs treated with and without antivenom.

## Materials and methods

### Study design and approval

Dogs bitten by *Vbb* and presenting to the first opinion emergency services at the small animal hospitals at the Norwegian University of Life Sciences (NMBU), Evidensia Oslo Dyresykehus, and Anicura Dyresykehus Oslo between April and October in 2017 and 2018 (two snake seasons) were assessed for enrolment in the study. Initial inclusion in the study required a diagnosis of snake bite based on history, presence of consistent clinical signs at presentation (fang marks, local signs of envenomation such as swelling and pain around the bite site, or systemic signs of envenomation such as lethargy, vomiting, collapse, and cardiac arrhythmias), and a minimum body weight of 10 kg. Exclusion criteria included any pre-existing disease and medications (other than prophylactic ectoparasitic treatment), presentation more than 24 hours after bite, a lack of clinical signs of envenomation within 12 hours after bite, and macroscopic haemolysis of plasma after centrifugation.

Twenty-eight privately-owned dogs, not previously bitten by *Vbb*, were recruited as healthy control dogs. Inclusion in the study required a minimum bodyweight of 10 kg, lack of significant acute or chronic disease, and no medication in the preceding two weeks. Healthy status was determined based on information obtained from the owner, lack of clinically significant abnormalities on physical examination, haematology and biochemistry profiles, and a C-reactive protein concentration within the reference interval.

This study was approved by the Ethical Committee for Approval of Studies with Animal Patients, at NMBU (14/04723 and 14/04723-48), and permitted by the Norwegian Food Safety Authority (Mattilsynet). Written owner consent was obtained for all dogs prior to inclusion in the study.

### Sample collection and processing

Blood was drawn from all envenomated dogs at five timepoints (T) after bite. T1 presentation, T2: 12 h, T3: 24 h, T4: 36 h and T5: 14 days. Blood was collected from the cephalic, saphenous, or jugular vein through an indwelling venous catheter or 21-G winged needle with extension. The first 1 mL of blood was discarded and a maximum of 6.4 mL of blood was thereafter collected into 3.2% sodium citrate tubes and centrifuged within 15 minutes at 2700 x g for 15 minutes, at room temperature. Top layer plasma was pooled, aliquoted into cryotubes and stored at -80°C for a maximum of 24 months prior to analysis. In healthy control dogs citrated plasma was collected, prepared and stored as for the envenomated dogs, and analysed within 4 months.

### Thrombin generation

Thrombin generation was measured using the CAT assay [24]. This assay has previously been used in dogs [25, 26] and is based on the cleavage of a fluorogenic thrombin substrate after triggering thrombin generation with calcium (Ca^2+^) and a reagent containing variable concentrations of TF and PLs. Eighty µL of citrated plasma was mixed with 20 µL reagent in a 96-well plate (Thermo Immulon 2HB plate, Thermo Scientific). To measure the overall thrombin-generating capacity of different counteracting constituents of plasma, for example coagulation factors and inhibitors such as antithrombin (AT) and tissue factor pathway inhibitor (TFPI), pro- and anticoagulant content of the venom, and presence of TF- and PS-exposing EVs, we used different reagents: PPP low reagent (1 pM TF, 4 µM PLs), PRP reagent (1 pM TF, minimal amounts of PLs), and no exogenous reagent (Tris-buffered saline (TBS, Sigma Aldrich (St. Louis, MO, USA)). To avoid excessively rapid initiation of thrombin generation, we diluted the PPP low reagent 1+1 with a reagent containing 4 µM PLs and no TF (MP-reagent) and the PRP reagent 1+1 with TBS, to obtain half the concentration of TF (but with sustained concentration of the PLs). Thrombin generation was initiated by automated addition of 20 µL FluCa buffer containing Ca^2+^ and a fluorogenic thrombin substrate (Z-gly-gly-arg-AMC, Thrombinoscope BV). Fluorescence was read for 60 min in a Fluoroscan Ascent microplate reader (Thermo Scientific, MA, USA), and the thrombin generation parameters lag time (time to initiation of thrombin generation) (LT), peak thrombin(the total amount of thrombin generated over time), and endogenous thrombin potential (the total amount of thrombin generated over time) (ETP) were calculated by the Thrombinoscope software (Thrombinoscope BV, Maastricht, the Netherlands). All reagents for the thrombin generation experiments were from Thrombinoscope BV (Maastricht, Netherlands).

### Procoagulant activity of phosphatidylserine (PS)-exposing extracellular vesicles (EVs)

Citrated plasma samples were thawed (10 min, 37°C) and lightly mixed before the activity of PS-exposing EVs was analysed with the Zymuphen MP activity kit (Hypen BioMed, Neuville-sur-Oise, France) according to the manufacturer’s protocol. Plasma samples had been thawed once previously. In samples with values above the highest standard (60 nM), the software extrapolated the amount of PS equivalents (nM), and the absolute values above 60 nM should therefore be interpreted with caution.

### Thrombin-antithrombin (TAT) complexes

Citrated plasma samples were thawed for 15 minutes at 37°C and gently mixed before the concentration of TAT complexes was determined with a sandwich enzyme immunoassay (Enzygnost®TAT micro, Siemens Healthcare Diagnostics) previously validated for canine plasma [27], according to the manufacturer’s protocol. Plasma samples had been thawed once previously. The standards included in the Enzygnost®TAT micro kit ranged from 2.0 to 60 µg/L. In samples with values above the highest standard, the software extrapolated the concentration of TAT, and the absolute values above 60 µg/L should therefore be interpreted with caution.

### In vitro effect of venom and antivenom

To investigate whether the *in vitro* effect of *Vbb* venom was pro- or anticoagulant, and whether the presence of antivenom might interfere in thrombin generation measurements, venom or antivenom were added to pooled plasma from three to five healthy controls, and thrombin generation was immediately initiated. Pooled *Vbb* venom (Siberian Serpentarium Ltd, Novosibirsk, Russia) was added at concentrations of 0, 12.5, 25 and 50 µg/mL, and antivenom (Viper Venom Antitoxin, SIS Biomed®, Warsaw, Poland) at concentrations of 0, 0.25, 0.5 and 1 U/mL.

### Statistical analysis

Creation of figures and statistical analysis were performed using commercially available statistical software packages (JMP Pro 14.3.0, SAS Institute Inc, Cary, NC and GraphPad Prism 8.3.1, GraphPad Software LLC, San Diego, CA). Data were visually assessed for normality. Most data were not normally distributed, thus median and range are reported throughout. Comparisons of LT, ETP, peak, PS equivalents and TAT complexes between the control group and each of the timepoints for the whole group of envenomated dogs and for dogs treated with and without antivenom, were performed using Steels test for multiple comparisons. Comparisons between dogs with and without antivenom treatment at each timepoint were made using Wilcoxon rank sum test. Fisher’s exact test was used to compare proportions of values in envenomated dogs that were above (peak, ETP) or below (LT) the control group values at presentation, between reagents.

Repeated measurements of outcome variables (thrombin generation, PS and TAT complexes) in envenomated dogs were analysed using a generalized linear mixed model with dog as a random effect, timepoint and antivenom treatment as fixed effects, using Stata SE 16.0 (Statcorp LLC, Texas, USA). Data was analysed using gaussian, gamma and negative binomial distributions along with the appropriate link function. The distribution showing the best fit, determined by an examination of the residuals as well as Akaike’s and Bayesian information criteria (AIC/BIC), was used to analyse the data. Pairwise comparisons were made between adjacent timepoints for the envenomated dogs. Post hoc Bonferroni correction was applied.

## Results

### Demographic data

The study population consisted of 28 of the 49 dogs originally enrolled. Four dogs were excluded for the following reasons: treatment with non-steroidal anti-inflammatory drugs (n = 1), presentation more than 24 hours after bite (n = 2) and no clinical signs of envenomation within 12 hours after bite (n = 1). Seventeen dogs, of which most had samples drawn from an indwelling venous catheter, were excluded due to macroscopic haemolysis at all sampling timepoints. Of the remaining 28 dogs, individual samples were excluded due to macroscopic haemolysis at the following timepoints: T3 (n = 1), T4 (n = 3) and T5 (n = 5). Samples were collected through an indwelling venous catheter in 6/28 dogs and via a winged needle with extension in 22/28 dogs. Further demographic data for the snake-bitten and healthy control dogs are presented in Table 1.

**Table 1:** Demographic data (median and range) for envenomated dogs and the control group.

	Envenomated dogs	Control group
**Number**	28	28
**Age (years)**	4.8 (0.3–13)	5 (0.8–12)
**Weight (kg)**	24.6 (13.5–43.0)	24.7 (11.0–50.0)
**Sex (male:female)(n)**	19: 9	15: 13
**Bite to presentation (hours)**	1.75 (0.25–8.50)	
**Bite location (head:limb) (n)**	22: 6	
Bite witnessed (n)^a^	17	
Antivenom treatment^b^ (yes:no) (n)	16: 12	
**Bite to antivenom (hours)**	4.75 (1.0–20.50)	

^a^snake, snakebite or fang marks witnessed. For the remaining dogs, a diagnosis was made based on history and compatible clinical signs.

^b^Equine F(ab’)_2_ antivenom intravenously (Viper Venom Antitoxin, SIS Biomed®, Warsaw, Poland).

### Physical examination and treatment

**Fig 1:**
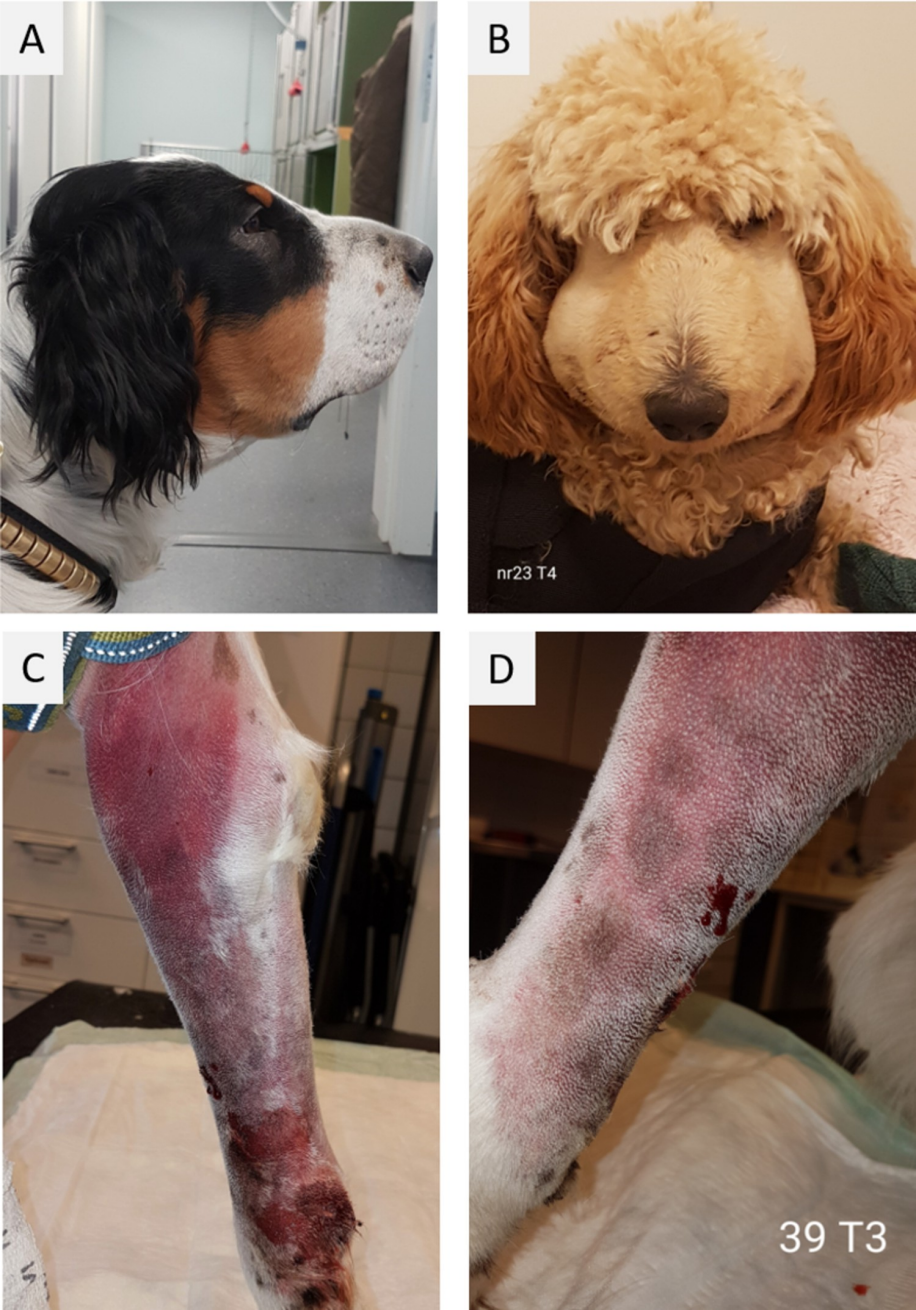
Examples of clinical findings in envenomated dogs. Facial swelling at presentation (T1) (A), extensive facial swelling 36 hours after bite (T4) (B), subcutaneous extravasation of blood 24 hours after bite (T3) (C) and continued bleeding from the bite wound in the same individual, 24 hours after bite (T3) (D).

Median sampling times were: T1: 3.5 hours (h) (range 0.5–7.5 h), T2: 12 h (9–16.5 h), T3: 24 h (22–25.5 h), T4: 36 h (32–39 h) and T5: 15 days (10–32 days). Physical examination findings were recorded at T1, T3 and T5 for envenomated dogs. Varying degrees of local swelling around the bite site were present in all dogs at T1. Swelling had resolved in one dog at T3 and in all but one dog at T5 in which a moderate swelling persisted. Haematomas and bleeding from the bite site were recorded in two dogs at T1. At T3, extensive subcutaneous extravasation of blood (bruising) and ecchymoses were recorded in two dogs. Examples of physical examination findings are presented in Fig 1. Overt clinical signs of thromboembolic disease were not observed in any dog at any timepoint.

Envenomated dogs were treated using a routine protocol including crystalloid fluid therapy (Ringer-acetate) intravenously (IV) at a standard rate of 4 mL/kg/hour and adjusted according to individual requirements, and buprenorphine (Vetergesic vet®, Ceva Santé Animale, France) at a dose of 0.01–0.02 mg/kg IV, intramuscularly (IM) or subcutaneously (SC) or methadone (Metadon, Norges Apotek, Norway) at a dose of 0.1–0.2 mg/kg IV or SC. Antivenom (Viper Venom Antitoxin, SIS Biomed®, Warsaw, Poland) was administered IV (n = 16) over one to two hours, at a dose of 500 units diluted in 250 mL 0.9% NaCl, within the first 24 hours after bite (Table 1). Indications for antivenom treatment included severe local or systemic signs or a lack of improvement or deterioration despite supportive treatment in dogs with mild local or systemic signs. Treatment decisions were made by the attending clinician.

### Missing data

When thrombin generation was initiated without an exogenous reagent, the thrombin generation curve did not reach the baseline in nine samples from envenomated dogs and for 15 control dogs. These ETP values are therefore missing in statistical analysis and peak values may be slightly overestimated in these individuals. Likewise, with PRP reagent, ETP is missing for three samples from envenomated dogs and for eight control dogs. For PPP reagent, LT was too fast to be registered by the software in five T1 samples from envenomated dogs. These LT values were arbitrarily set to 1.33 minutes, i.e. the lowest LT registrable by the software, and included in statistical analysis. In these five cases peak thrombin and ETP could not be calculated due to the lack of a starting point and thus are missing. Statistical comparisons between antivenom and non-antivenom treated dogs at T1 and between antivenom treated dogs at T1 and controls were not made due to only two dogs in the antivenom treated group.

### Thrombin generation in envenomated dogs and healthy controls

**Fig 2:**
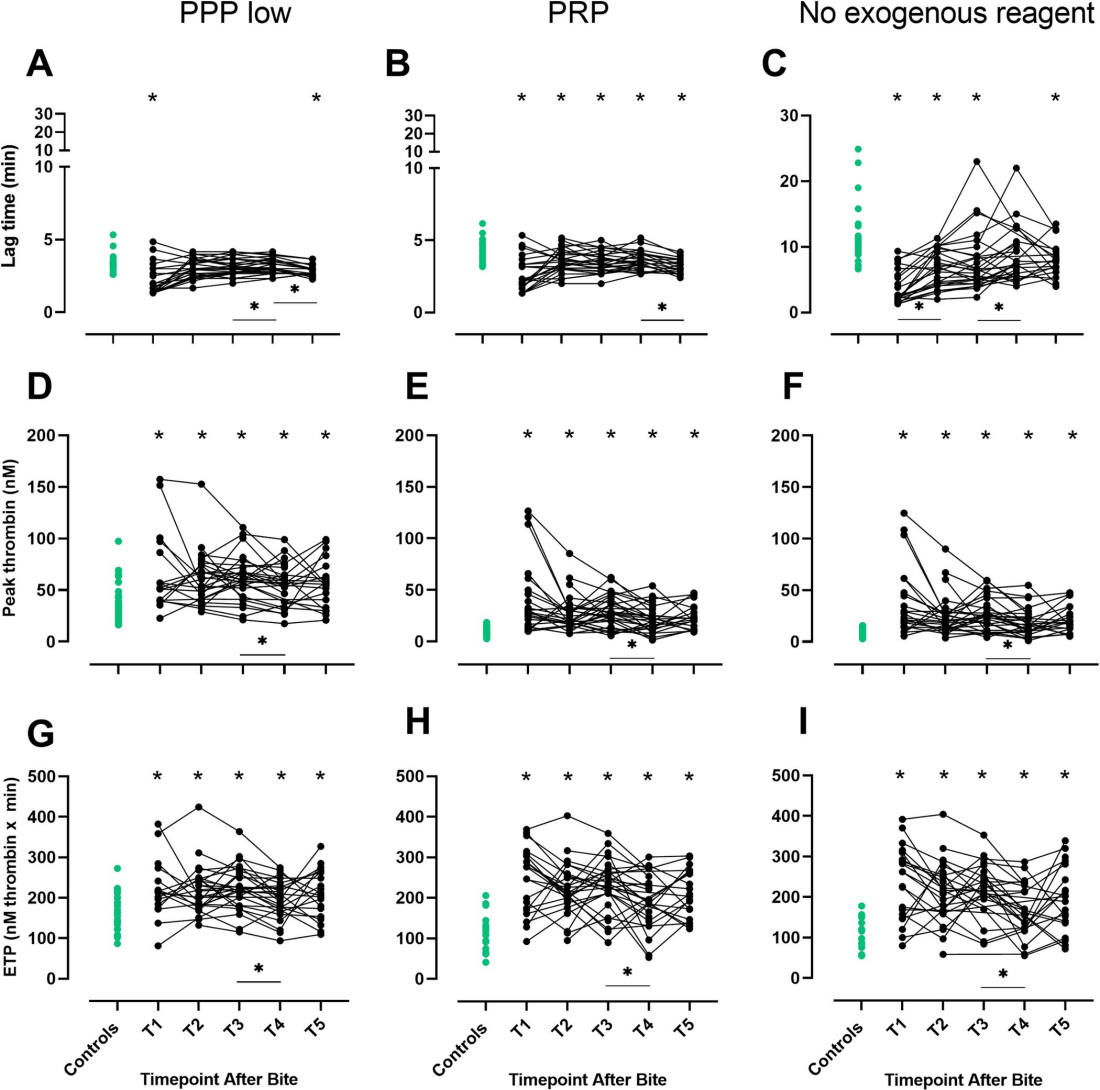
Thrombin generation parameters in envenomated dogs and controls. Lag time, peak thrombin and endogenous thrombin potential (ETP) measured by calibrated automated thrombography with three reagents, PPP low, PRP and no exogenous reagent, in envenomated dogs at five timepoints after bite (black dots) and healthy controls (green dots). T1 = presentation, T2 = 12 hours, T3 = 24 hours, T4 = 36 hours and T5 = 15 days after bite. * indicates P < 0.05 for comparisons between envenomated dogs at each time point and the control group. 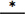 indicates a significant change between timepoints for envenomated dogs (P < 0.05).

Overall, thrombin generation was increased at most time-points in envenomated dogs compared to controls. With PPP low reagent, LT was significantly shorter at T1 and T5 in envenomated dogs compared to controls. In the envenomated dogs, LT increased significantly from T3 to T4 and then decreased from T4 to T5. With PRP reagent, LT was significantly shorter at all timepoints (T1-T5) in envenomated dogs compared to controls and decreased significantly between T4 and T5. For no exogenous reagent, LT was shorter at T1-T3 and T5 in envenomated dogs compared to controls, with significant increases occurring from T1 to T2 and from T3 to T4 (Fig 2A–2C, S1 Table). With all reagents, including no exogenous reagent, peak and ETP were significantly higher at all timepoints for envenomated dogs than for controls, and decreased significantly between T3 and T4 (Fig 2D–2I, S1 Table).

**Effect of different thrombin generation reagents.** We also explored which reagent differentiated best between the control group and envenomated dogs at presentation. The magnitude of difference in thrombin generation between control dogs and envenomated dogs, differed according to the reagent used. When thrombin generation was initiated with PRP or no exogenous reagent (i.e. both without PLs), proportions of envenomated dogs with peak thrombin values above the highest control value were significantly higher, compared to when thrombin generation was initiated with the phospholipid-containing PPP low reagent (both P = 0.003, Table 2). For ETP, a similar pattern was observed, although proportions of dogs with values greater than the maximum control value did not differ significantly between the reagents after Bonferroni correction (both P = 0.07, Table 2). For LT, the highest proportion of envenomated dogs with LT below the lowest control value was found with no exogenous reagent added, although proportions did not differ significantly between reagents.

**Table 2:** Proportion of envenomated dogs with values for lag time (LT) below the minimum value for the control group, and peak thrombin and endogenous thrombin potential (ETP) values above the maximum value for the control group, at presentation, for each reagent (PPP low, PRP and no exogenous reagent).

	PPP low	PRP	No exogenous reagent
**LT**	68% (15/22)	68% (15/22)	77% (17/22)
**Peak thrombin**	18% (3/17)	73% (16/22)*	73% (16/22)*
**ETP**	24% (4/17)	62% (13/21)	62% (13/21)

* indicates a significant difference (P < 0.05) compared to PPP low, after Bonferroni correction.

### Thrombin generation in envenomated dogs with and without antivenom treatment

**Fig 3:**
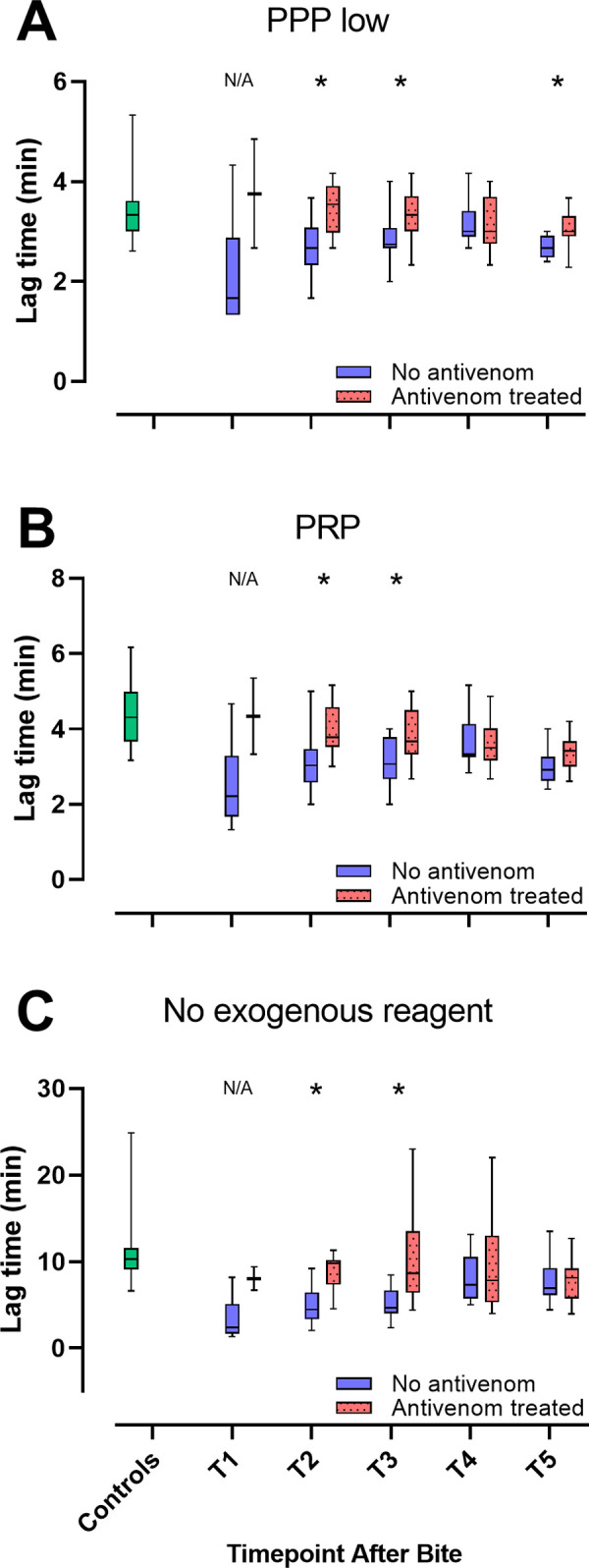
Lag time in dogs treated with and without antivenom. Box and Whisker plots for lag time with three different reagents (PPP low (A), PRP (B) and no exogenous reagent (C)), in dogs treated with (red) and without (blue) antivenom, and healthy controls (green). T1 = presentation, T2 = 12 hours, T3 = 24 hours, T4 = 36 hours and T5 = 15 days after bite.* indicates a significant difference (P < 0 .05) between treatment groups at a given timepoint. Statistical analysis was not performed at T1 due to a low number of antivenom treated dogs (n = 2). N/A = not analysed.

Thrombin generation differed significantly between dogs treated with and without antivenom, an effect best observed with LT. With all reagents, LT was significantly shorter in dogs not treated with antivenom compared to antivenom treated dogs at T2 and T3 (all P values ≤ 0.03, Fig 3A–3C). In the antivenom treated dogs, LT did not differ significantly from the control group at these timepoints with any of the reagents (all P values ≥ 0.07). Statistical analysis was not performed at T1 due to only two dogs treated with antivenom at this timepoint. For peak thrombin and ETP, there were no differences between dogs with and without antivenom-treatment, except for at T4 with PPP low reagent, where peak was significantly higher for the antivenom treated dogs compared to dogs not treated with antivenom (P = 0.03, S1A Fig), and at T2 with no exogenous reagent, where ETP was significantly higher in dogs that did not receive antivenom compared to those that did (P = 0.03, S1F Fig).

### Phosphatidylserine (PS) equivalents

**Fig 4:**
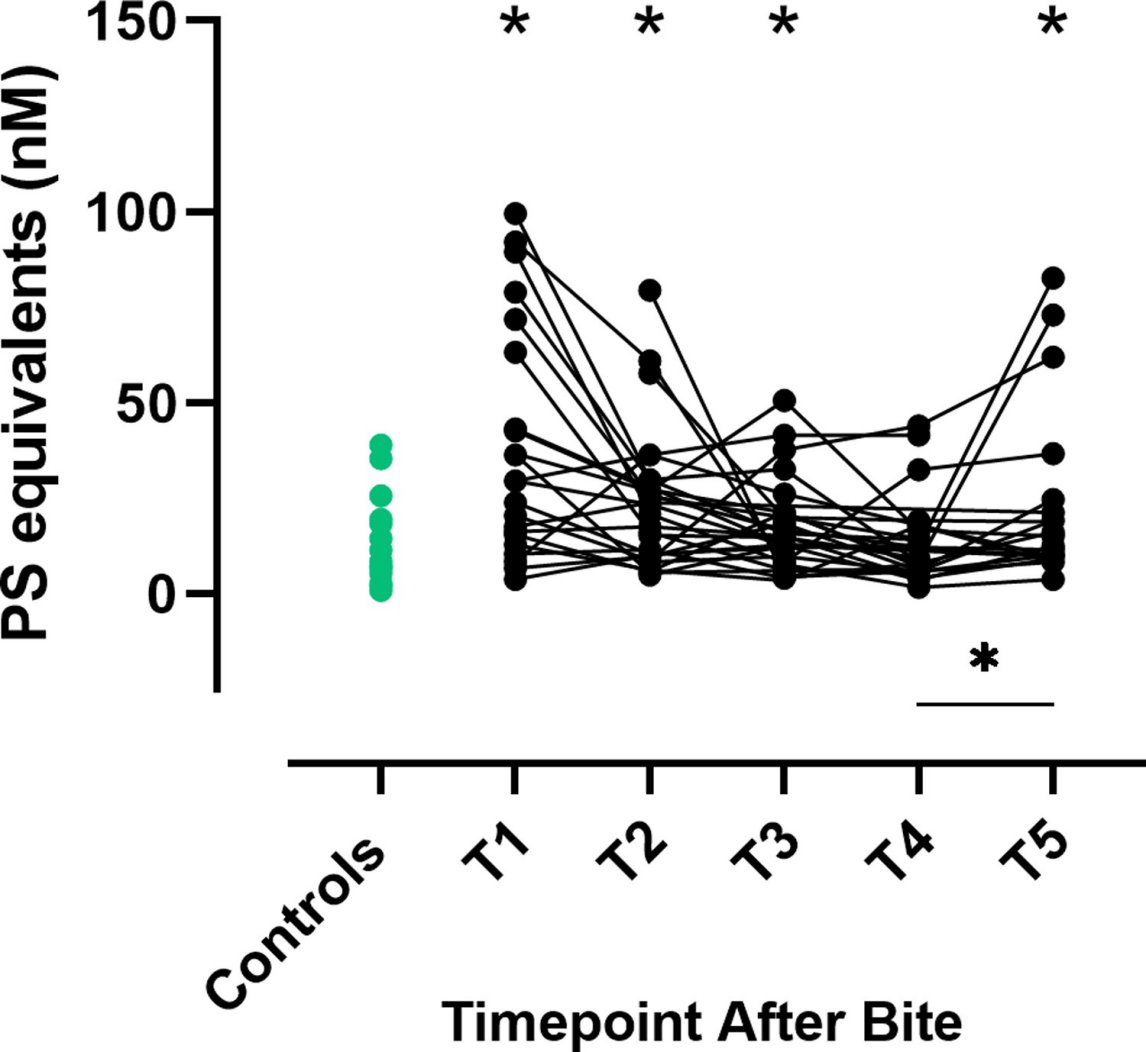
Phosphatidylserine equivalents in envenomated dogs and controls. Phosphatidylserine (PS) equivalents in envenomated dogs at five timepoints after bite (black dots) and controls (green dots). T1 = presentation, T2 = 12 hours, T3 = 24 hours, T4 = 36 hours and T5 = 15 days after bite.* indicates P < 0.05 for comparisons between envenomated dogs at each time point and the control group. 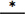 indicates a significant change between timepoints for envenomated dogs (P < 0.05).

The procoagulant activity of PS-expressing EVs was measured as PS equivalents. Compared to the control group, PS equivalents were significantly higher in envenomated dogs at T1-T3 and T5 (Fig 4, S2 Table). Levels of PS equivalents increased significantly from T4 to T5 (P = 0.024, Fig 4). PS equivalents were significantly higher at T3 in dogs not treated with antivenom compared to those that received antivenom (P = 0.02, S2 Fig).

### Thrombin-antithrombin (TAT) complexes

**Fig 5:**
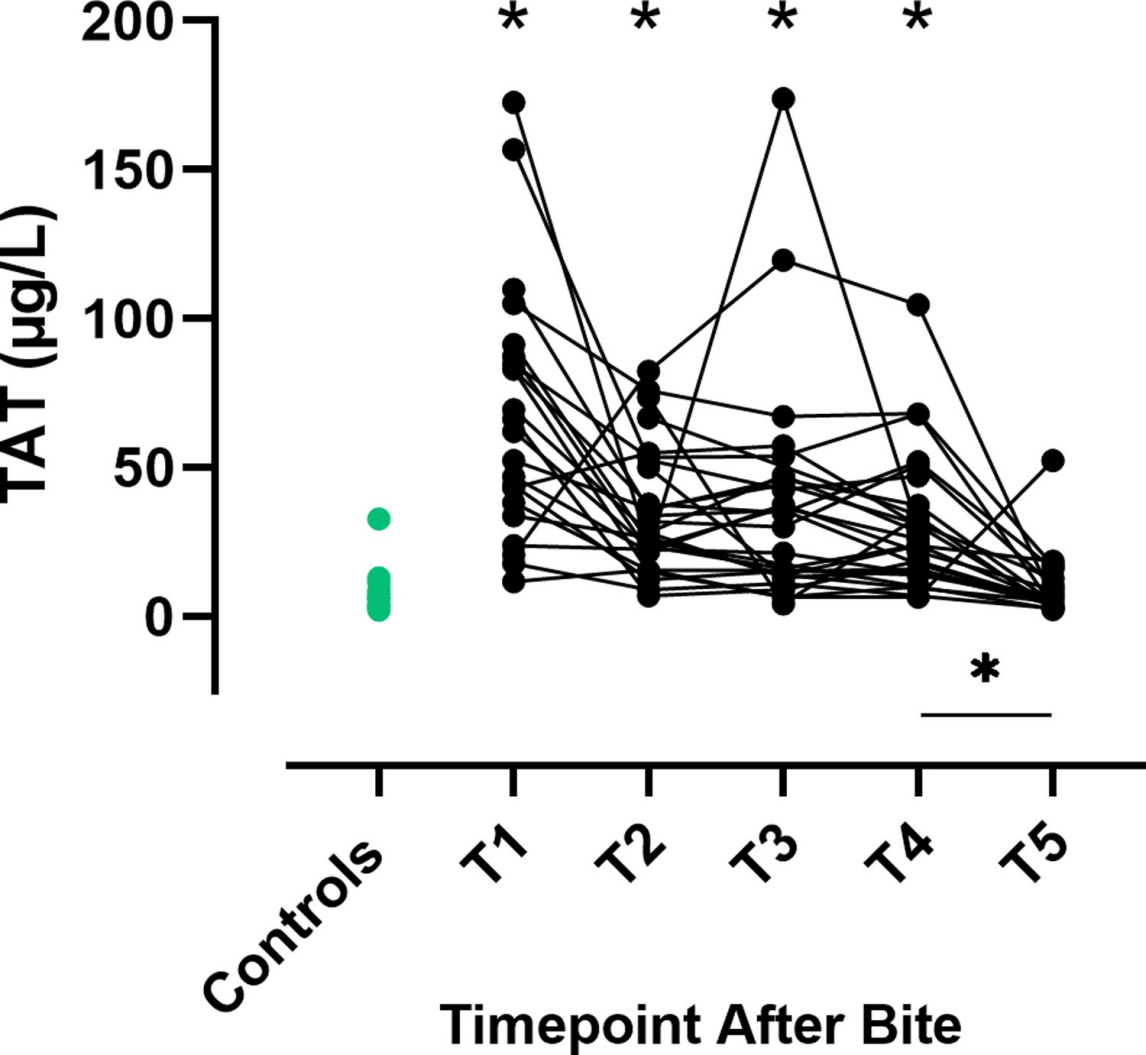
Thrombin-antithrombin complexes in envenomated dogs and controls. Concentrations of thrombin-antithrombin (TAT) complexes in envenomated dogs at five timepoints after bite (black dots) and controls (green dots). T1 = presentation, T2 = 12 hours, T3 = 24 hours, T4 = 36 hours and T5 = 15 days after bite.* indicates P < 0.05 for comparisons between envenomated dogs at each time point and the control group. 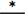 indicates a significant change between timepoints for envenomated dogs (P < 0.05).

Compared to the control group, concentrations of TAT complexes were significantly increased at T1-T4 (Fig 5, S2 Table). In envenomated dogs, a significant decrease in TAT was observed between T4 and T5 (P < 0.0001, Fig 5), and at T5 the TAT concentration was similar to the controls. TAT values were not significantly different between treatment groups except at T3 where TAT was higher in the non-antivenom treated dogs (P = 0.002, S3 Fig).

### In vitro effect of venom and antivenom

Overall, antivenom did not appear to affect the thrombin generation curves (S4 Fig). When plasma was incubated with venom, thrombograms were unable to be generated due to rapid initiation of thrombin generation, even at the lowest venom concentration, indicating that the venom has procoagulant characteristics.

## Discussion

In this longitudinal study we measured the global coagulation status of dogs envenomated by *Vbb*, from presentation to median 15 days after bite. At presentation, the envenomated dogs were hypercoagulable compared to the healthy control dogs, as demonstrated by increased thrombin generation, TAT complexes and PS equivalents. Hypercoagulability gradually decreased but persisted at day 15, except for TAT complexes. LT was normalised in dogs treated with antivenom, suggesting that treatment may counteract hypercoagulability.

The most pertinent finding was that the envenomated dogs were hypercoagulable at presentation compared to the healthy control dogs. In contrast to our study, most previous studies using PT, INR and APTT are indicative of hypocoagulability after *Vbb* envenomation in humans and dogs [4, 12, 19]. Prolongation of PT and APTT is used in human and veterinary medicine as a reliable indicator of envenomation in cases of suspected snake bite, although normal values do not exclude the possibility of envenomation [28]. One study reported short INR values at presentation in 2/119 humans envenomated by *Vbb*, indicating hypercoagulability [4, 29]. Short PT and APTT values were also reported in one *in vitro* study of *Vbb* venom [30]. Due to the inherent insensitivity of PT and APTT to detect a procoagulant state, hypercoagulability after *Vbb* bite has likely been underestimated in previous studies using these routine clotting analyses [31].

Clinical findings of thrombosis and bleeding have previously been reported in humans and dogs envenomated by Vbb [4, 10–12]. Hypercoagulable individuals may be at risk of thrombosis [32, 33], but no dogs in this study had clinically suspected thromboembolism. Histopathological examination of organs likely to be affected by microthrombi, such as the kidneys, was not performed due to ethical considerations and a lack of clinical indication. Thus, the clinical significance of the procoagulant state demonstrated in this study is unclear. Despite the hypercoagulability demonstrated in our study, four dogs had clinical evidence of bleeding. Neither the platelet or fibrinolytic contributions to haemostasis are evaluated using CAT, and we therefore cannot rule out platelet dysfunction or enhanced fibrinolysis as a cause of bleeding in these dogs.

Tissue injury and ongoing inflammation after snakebite [34, 35] may contribute to the release of procoagulant EVs [18]. The discrepancy in peak thrombin levels between healthy controls and envenomated dogs at presentation was greater when thrombin generation was phospholipid-dependent (i.e. using PRP reagent or no exogenous reagent), compared to phospholipid-independent (using PPP low reagent), indicating that PL-rich EVs may contribute to increased thrombin generation in envenomated dogs. This is also supported by the increased procoagulant activity of PS-exposing EVs (PS equivalents) in envenomated dogs at most timepoints, compared to the control group.

The repeated measurements in our study enabled us to detect a persistent hypercoagulability not previously described after *Vbb* envenomation. We demonstrated that *Vbb* venom was procoagulant *in vitro* but data regarding the distribution and half-life kinetics of *Vbb* venom have not been published. However, the venom composition of *Vipera aspis (V*. *aspis)* is similar to that of *Vbb* [36] and it is reasonable to assume they show similar elimination kinetics. The half-life of *V*. *aspis* venom in humans is eight hours [37], but case reports pertaining to other snake species have described a suspected depot effect of venom, allowing venom components to persist for weeks after envenomation [38, 39]. Thus, the venom could possibly contribute to the prolonged hypercoagulability observed in our study. Despite the persistent hypercoagulability in the plasma of dogs 15 days after bite in our study, we observed both clinical improvement and normalised TAT complex concentrations at this timepoint, indicating that there might be a difference between potential and actual thrombin generation in these dogs.

Clinical studies in rabbits and mice describe neutralisation of *Vbb* venom by antivenom [40, 41], including a reduction in local haemorrhagic effects, but there are no studies focusing on laboratory analyses of coagulation in the context of antivenom efficacy after *Vbb* envenomation. However, *in vitro* thromboelastography measurements in canine whole blood showed early hypercoagulable changes when exposed to venom of another viper, *Vipera palastinae*, that were annulled by addition of specific antivenom [42]. Antivenom treated dogs in our study had LTs similar to those of the control group, and significantly longer LTs than the non-antivenom treated dogs during the first 24 hours after bite. Whilst our study design does not allow us to imply causation, this is nevertheless a very interesting finding which warrants further investigation especially in light of the current lack of evidence supporting the use of antivenom in treating dogs envenomated by *Vbb* [43]. Significant differences between the antivenom treated group and the non-treated group were almost exclusively observed with LT, and not peak or ETP, the reason for which is unknown. We cannot exclude that a confounding effect such as severity of envenomation, or coagulation factor depletion may have been present in the envenomated dogs selected for treatment with antivenom. Selective depletion of coagulation factors important in the initiation phase of coagulation could potentially result in longer LTs without affecting peak and ETP. A neutralising effect of antivenom on venom components that specifically influence the initiation phase of thrombin generation is another possible explanation. A randomized control trial (RCT) of antivenom efficacy and further characterisation of the coagulation factor activities in envenomated dogs could clarify this question.

Phospholipid-dependent thrombin generation measurement, and especially the LT, may also be interesting to evaluate as markers to help confirm whether a dog has received a venomous bite or not. This could be particularly useful considering that fang marks may be absent in up to 50% of envenomated dogs [44] and it is estimated that up to 30% of *Vbb* bites do not contain venom (‘dry bites’) [45]. Early confirmation or exclusion of *Vbb* envenomation could be of substantial clinical benefit considering the cost of treatment and potential for attenuation of venom-induced coagulation disturbances [42, 46].

There are some limitations to this study. Only two dogs had received antivenom treatment at T1 and statistical comparisons were therefore not made between dogs treated with and without antivenom at this timepoint. Further studies focusing on both the effects of antivenom treatment at this early timepoint and timing of treatment relative to time of bite, would be interesting. A lower bodyweight to venom ratio is possible in smaller dogs and could result in more severe envenomation effects [44]. Due to the volumes of blood collected in this study, only dogs over 10 kg bodyweight were included. We therefore cannot rule out the possibility of a selection bias resulting in some smaller, more severely affected dogs being excluded from this study. The use of a snakebite severity scoring system in future studies could be useful to assess differences in severity of envenomation between dogs treated with and without antivenom. A large number of samples were excluded due to haemolysis since this is known to influence thrombin generation results [47]. However, the majority of excluded samples were from dogs sampled through an indwelling venous catheter and thus haemolysis was suspected to be due to sampling technique rather than an *in vivo* effect. A number of PS-equivalent and TAT complex values were above the limit of detection for the assay. Although these values can be considered high, exact concentrations in this range are therefore uncertain. Citrated plasma samples were centrifuged only once, which may lead to residual platelets that fragment during a freeze-thaw cycle. PS-exposing EVs have been found to increase after a single freeze-thaw cycle [48] which may increase phospholipid-dependent thrombin generation and PS equivalents [49]. We cannot exclude that an extra freeze-thaw cycle may have increased PS equivalents further. Double centrifugation (2x 2500 g for 15 minutes) is recommended to minimise these effects [50]. Finally, dogs and humans might respond differently to snake venom coagulotoxins [51]. Thus, it is unclear to what extent findings in this study might be applicable to human *Vbb* envenomation.

In conclusion, repeated measurements of thrombin generation in plasma indicate a procoagulant state in dogs bitten by *Vbb* already at presentation that, although decreasing, is still present 15 days after bite. Whether the hypercoagulable state after *Vbb* envenomation has clinical implications remains to be determined. The thrombin generation parameter LT in dogs treated with antivenom reached similar levels to that of the healthy controls, indicating that antivenom treatment might counteract hypercoagulability. Thrombin generation measurement might serve both as a diagnostic and as a monitoring tool after *Vbb* envenomation and may be a suitable marker in future studies of antivenom efficacy.

## Supporting information

S1 Table Thrombin generation parameters for three different reagents (PPP low, PRP and no exogenous reagent) in envenomated dogs at five timepoints (T1 = presentation, T2 = 12 h, T3 = 24 h, T4 = 36 h, T5 = 15 days) after bite, and controls. Values are given as median (range) for lag time, peak thrombin and endogenous thrombin potential (ETP). P-value1 represents comparisons to controls. P-value2 represents comparisons to the subsequent timepoint. Significant P-values (P < 0.05) are in bold.
(DOCX)

S2 Table Phosphatidylserine (PS) equivalents and thrombin-antithrombin (TAT) complex concentrations in envenomated dogs at five timepoints after bite (T1 = presentation, T2 = 12h, T3 = 24h, T4 = 36 h, T5 = 15 days), and controls. Values are given as median (range). P-value1 represents comparisons to controls. P-value2 represents comparisons to the subsequent timepoint. Significant P-values are in bold.
(DOCX)

S1 Fig Thrombin generation parameters in dogs treated with and without antivenom. Box and whisker plots for peak thrombin (A-C) and endogenous thrombin potential (ETP) (D-F) with three different reagents (PPP low, PRP and no exogenous reagent), in dogs treated with (red) and without (blue) antivenom, and controls (green). T1 = presentation, T2 = 12 hours, T3 = 24 hours, T4 = 36 hours and T5 = 15 days after bite. * indicates a significant difference (P < 0 .05) between treatment groups at a given time point. Statistical analysis was not performed at T1 due to a low number of antivenom treated dogs (n = 2). N/A = not analysed.
(TIF)

S2 Fig Phosphatidylserine (PS) equivalents in dogs treated with and without antivenom. Box and Whisker plots for PS equivalents in dogs treated with (red) and without (blue) antivenom, and controls (green). T1 = presentation, T2 = 12 hours, T3 = 24 hours, T4 = 36 hours and T5 = 15 days after bite. * indicates a significant difference (P < 0 .05) between treatment groups at a given time point. Statistical analysis was not performed at T1 due to a low number of antivenom-treated dogs (n = 2). N/A = not analysed.
(TIF)

S3 Fig Thrombin-antithrombin (TAT) complexes in dogs treated with and without antivenom. Box and Whisker plots for TAT complexes in dogs treated with (red) and without (blue) antivenom, and controls (green). T1 = presentation, T2 = 12 hours, T3 = 24 hours, T4 = 36 hours and T5 = 15 days after bite. * indicates a significant difference (P < 0 .05) between treatment groups at a given time point. Statistical analysis was not performed at T1 due to a low number of antivenom-treated dogs (n = 2). N/A = not analysed.
(TIF)

S4 Fig Thrombograms with different concentrations of antivenom. Thrombograms with three different reagents (PPP low, PRP and no exogenous reagent) with antivenom at concentrations of 0 (black), 0.25 (red), 0.5 (blue) and 1 U/mL (green).
(TIF)

S1 Data (XLSX)
