# Supplementary material for: Persistent hypercoagulability in dogs envenomated by the European adder (Vipera berus berus)
Source: PLoS One. 2022 Feb 18;17(2):e0263238. doi: 10.1371/journal.pone.0263238 (PMC8856559; doi:10.1371/journal.pone.0263238)
Supplement: S1 Table — Values are given as median (range) for lag time, peak thrombin and endogenous thrombin potential (ETP). P-value1 represents comparisons to controls. P-value2 represents comparisons to the subsequent timepoint. Significant P-values (P < 0.05) are in bold. (DOCX) [file pone.0263238.s001.docx]

|  | | **Lag time (min)** | | **Peak (nM thrombin)** | | **ETP (nM thrombin x min)** | |
| --- | --- | --- | --- | --- | --- | --- | --- |
|  |  | Median  (range) | P-value^1^  P-value^2^ | Median  (range) | P-value^1^  P-value^2^ | Median  (range) | P-value^1^  P-value^2^ |
| PPP low | Controls | 3.3  (2.6-5.3) |  | 33  (16-97) |  | 144  (86-272) |  |
|  | T1 | 1.7  (1.3-4.9) | **0.0006**  > 0.99 | 52  (23-157) | **0.008**  > 0.99 | 213  ( 81-382) | **0.008**  > 0.99 |
|  | T2 | 3  (1.7-4.2) | 0.45  > 0.99 | 61  (29-153) | **0.0002**  > 0.99 | 217  (132-424) | **0.0002**  > 0.99 |
|  | T3 | 3.0  (2.0-4.2) | 0.7  **0.02** | 59  (21-111) | **0.0007**  **0.03** | 221  (115-364) | **0.0001**  **0.002** |
|  | T4 | 3.0  (2.3-4.2) | 0.83  **< 0.0001** | 57  (18-99) | **0.01**  > 0.99 | 202  (94-274) | **0.018**  > 0.99 |
|  | T5 | 3.0  (2.3-3.7) | **0.02** | 56  (21-99) | **0.027** | 208  (110-327) | **0.013** |
| PRP | Controls | 4.3  (3.2-6.2) |  | 9  (3-19) |  | 114  (41 – 206) |  |
|  | T1 | 2.3  (1.3- 9.4) | **< 0.0001**  > 0.99 | 29  (10- 126) | **< 0.0001**  0.31 | 276  (92-369) | **< 0.0001**  0.73 |
|  | T2 | 3.5  (2.0-5.2) | **0.007**  0.56 | 22.  (8-85) | **< 0.0001**  > 0.99 | 213  (95-403) | **< 0.0001**  > 0.99 |
|  | T3 | 3.3  (2.0-5.0) | **0.003**  0.23 | 27  (6-62) | **< 0.0001**  **0.008** | 233  (90-359) | **< 0.0001**  **0.04** |
|  | T4 | 3.4  (2.7-5.2) | **0.006**  **< 0.0001** | 20  (1-54) | **0.003**  0.56 | 189  (53-301) | **0.003**  0.4 |
|  | T5 | 3.2  (2.4-4.2) | **< 0.0001** | 22  (9-47) | **< 0.0001** | 206  (12- 303) | **< 0.0001** |
| No exogenous reagent | Controls | 10.3  (6.6-24.9) |  | 8  (2-16) |  | 97.9  (55-178) |  |
|  | T1 | 2.4  (1.3-4.8) | **< 0.0001**  **0.014** | 25  (5-125) | **< 0.0001**  0.12 | 30  (4-100) | **0.0008**  0.05 |
|  | T2 | 6.8  (2.0-11.3) | **< 0.0001**  > 0.99 | 21  (4-90) | **< 0.0001**  > 0.99 | 215  (59-405) | **0.0003**  > 0.99 |
|  | T3 | 6.6  (2.3-23.0) | **0.001**  **0.005** | 24  (4-60) | **< 0.0001**  **0.023** | 229  (84-353) | **0.0002**  **< 0.0001** |
|  | T4 | 7.3  (4.0-22) | 0.06  > 0.99 | 15  (1-55) | **0.03**  0.50 | 161  (56-287) | **0.04**  0.18 |
|  | T5 | 7.8  (4.0-13.5) | **0.003** | 19  (5-48) | **0.0004** | 195.5  (72-339) | **0.009** |
