# Supplementary material for: Persistent hypercoagulability in dogs envenomated by the European adder (Vipera berus berus)
Source: PLoS One. 2022 Feb 18;17(2):e0263238. doi: 10.1371/journal.pone.0263238 (PMC8856559; doi:10.1371/journal.pone.0263238)
Supplement: S2 Table — Values are given as median (range). P-value1 represents comparisons to controls. P-value2 represents comparisons to the subsequent timepoint. Significant P-values are in bold. (DOCX) [file pone.0263238.s002.docx]

|  | **PS equivalents (nM)** | | **TAT complexes (µg/L)** | |
| --- | --- | --- | --- | --- |
|  | Median  (range) | P-value^1^  P-value^2^ | Median  (range) | P-value^1^  P-value^2^ |
| Controls | 7  (1-39) |  | 6  (2-33) |  |
| T1 | 30  (4-100) | **0.0001**  0.3 | 64  (12-173) | **< 0.0001**  > 0.99 |
| T2 | 21  (5-80) | **0.003**  0.79 | 28  (7-82) | **< 0.0001**  > 0.99 |
| T3 | 14  (4-51) | **0.03**  0.28 | 33  (4-174) | **< 0.0001**  > 0.99 |
| T4 | 8  (2-44) | 0.66  **0.02** | 29  (7-105) | **< 0.0001**  **< 0.0001** |
| T5 | 14  (4-83) | **0.007** | 5  (2-52) | 0.99 |
