# Supplementary figures and images for: Persistent hypercoagulability in dogs envenomated by the European adder (Vipera berus berus)
Source: PLoS One. 2022 Feb 18;17(2):e0263238. doi: 10.1371/journal.pone.0263238 (PMC8856559; doi:10.1371/journal.pone.0263238)

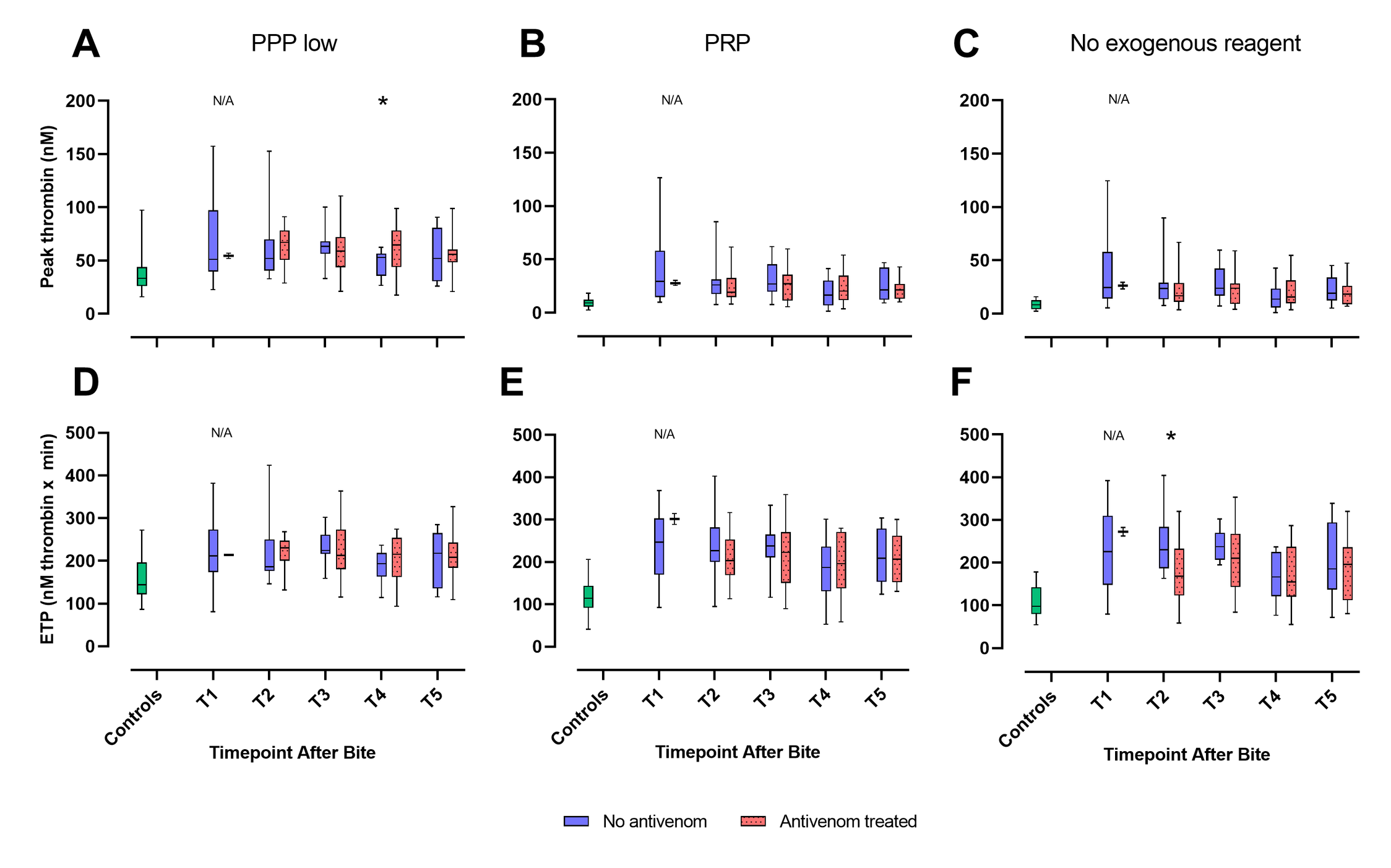

Supplement: S1 Fig — Box and whisker plots for peak thrombin (A-C) and endogenous thrombin potential (ETP) (D-F) with three different reagents (PPP low, PRP and no exogenous reagent), in dogs treated with (red) and without (blue) antivenom, and controls (green). T1 = presentation, T2 = 12 hours, T3 = 24 hours, T4 = 36 hours and T5 = 15 days after bite. * indicates a significant difference (P < 0 .05) between treatment groups at a given time point. Statistical analysis was not performed at T1 due to a low number of antivenom treated dogs (n = 2). N/A = not analysed. (TIF) [file pone.0263238.s003.tif]

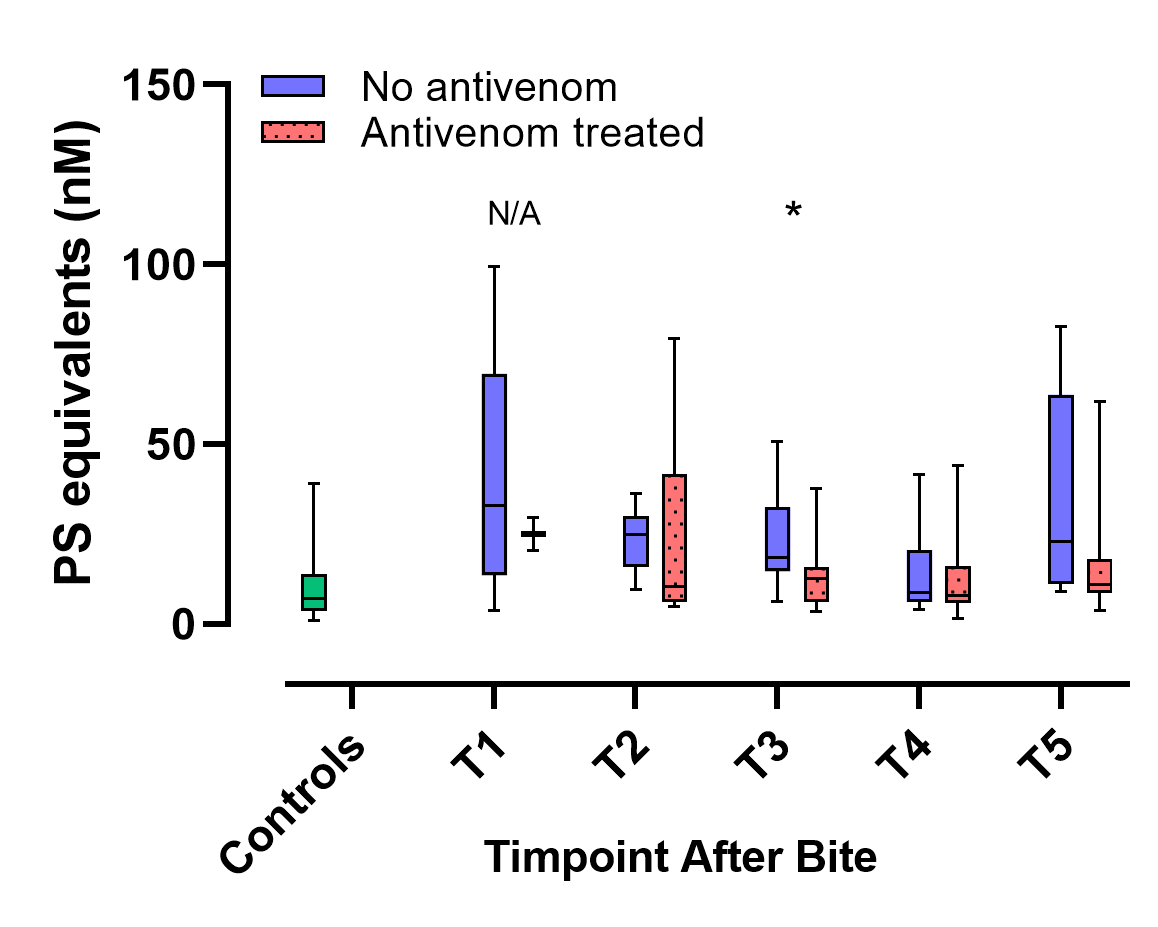

Supplement: S2 Fig — Box and Whisker plots for PS equivalents in dogs treated with (red) and without (blue) antivenom, and controls (green). T1 = presentation, T2 = 12 hours, T3 = 24 hours, T4 = 36 hours and T5 = 15 days after bite. * indicates a significant difference (P < 0 .05) between treatment groups at a given time point. Statistical analysis was not performed at T1 due to a low number of antivenom-treated dogs (n = 2). N/A = not analysed. (TIF) [file pone.0263238.s004.tif]

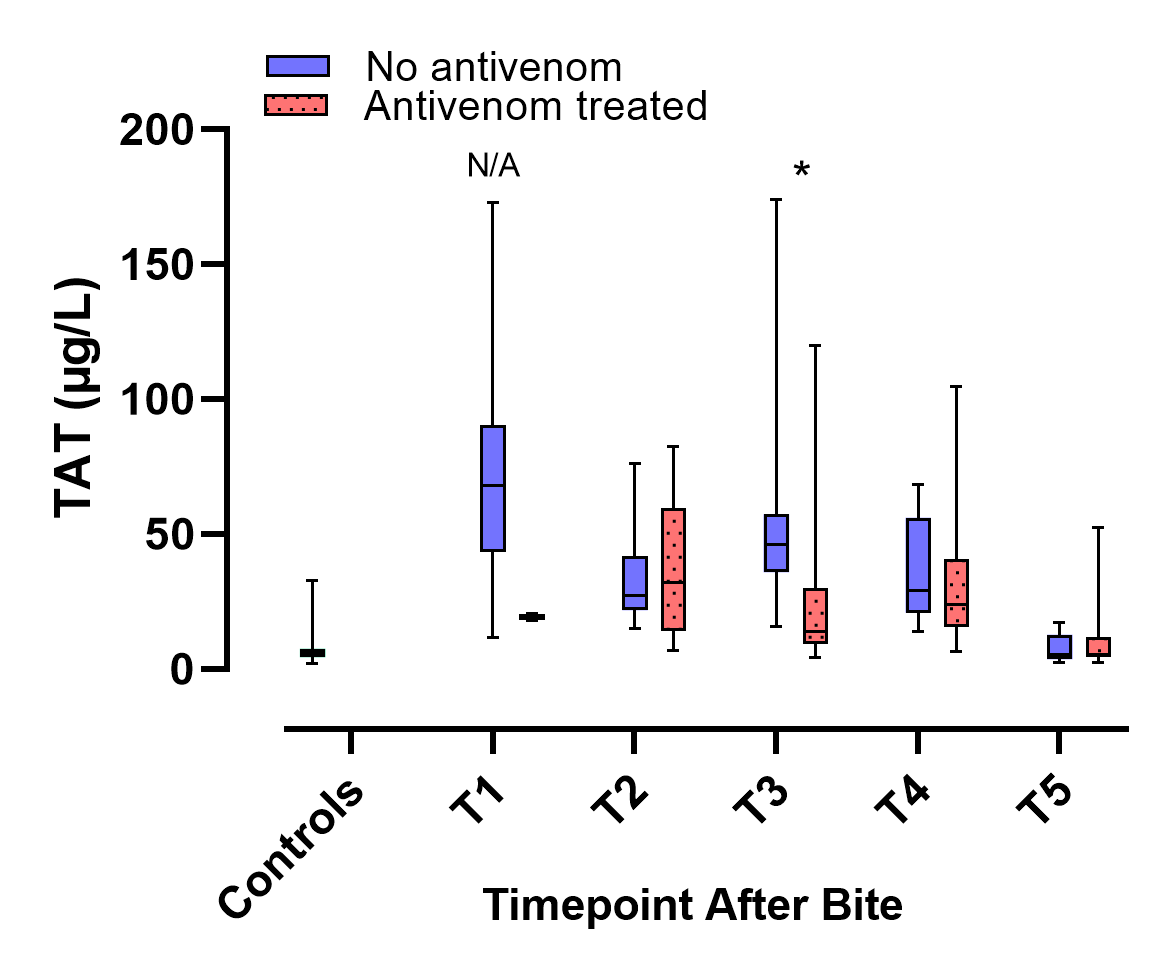

Supplement: S3 Fig — Box and Whisker plots for TAT complexes in dogs treated with (red) and without (blue) antivenom, and controls (green). T1 = presentation, T2 = 12 hours, T3 = 24 hours, T4 = 36 hours and T5 = 15 days after bite. * indicates a significant difference (P < 0 .05) between treatment groups at a given time point. Statistical analysis was not performed at T1 due to a low number of antivenom-treated dogs (n = 2). N/A = not analysed. (TIF) [file pone.0263238.s005.tif]

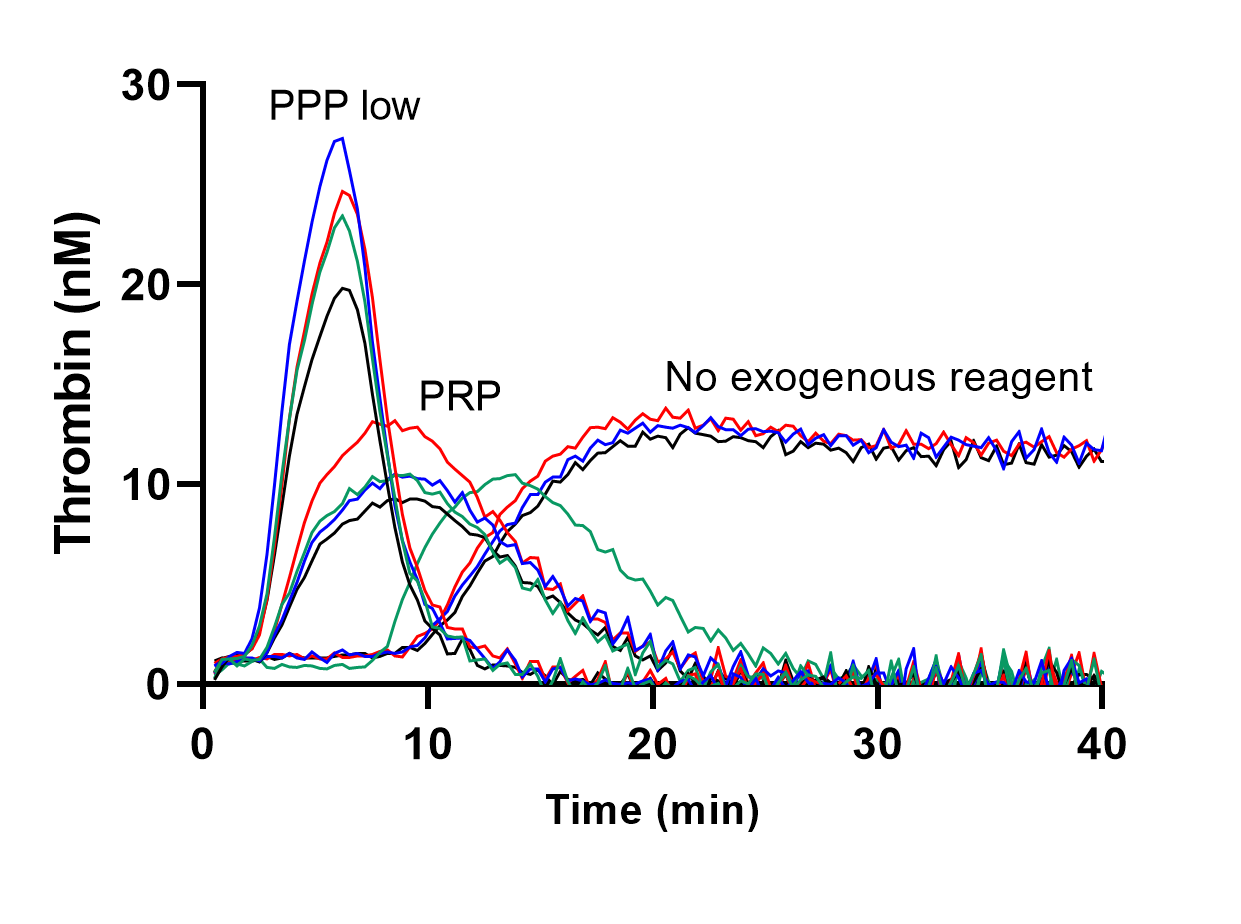

Supplement: S4 Fig — Thrombograms with three different reagents (PPP low, PRP and no exogenous reagent) with antivenom at concentrations of 0 (black), 0.25 (red), 0.5 (blue) and 1 U/mL (green). (TIF) [file pone.0263238.s006.tif]
